# Supplementary material for: Prognostic nutritional index (PNI) as an influencing factor for in-hospital mortality in patients with stroke-associated pneumonia: a retrospective study
Source: PeerJ. 2025 Feb 26;13:e19028. doi: 10.7717/peerj.19028 (PMC11871890; doi:10.7717/peerj.19028)
Supplement: Supplemental Information 1 [file peerj-13-19028-s001.docx]

STROBE Statement—checklist of items that should be included in reports of observational studies

|  | Item No. | Recommendation | Page  No. | Relevant text from manuscript |
| --- | --- | --- | --- | --- |
| **Title and abstract** | 1 | (*a*) Indicate the study’s design with a commonly used term in the title or the abstract | Page1 | Prognostic nutritional index (PNI) as an influencing factor for in-hospital mortality in patients with stroke-associated pneumonia: A retrospective study |
|  |  | (*b*) Provide in the abstract an informative and balanced summary of what was done and what was found | Page2 | PNI levels in SAP patients were associated with the risk of in-hospital mortality in patients, and increased PNI levels exerted an independent protective effect on the short-term prognosis of patients. |
| Introduction | | | |  |
| Background/rationale | 2 | Explain the scientific background and rationale for the investigation being reported | Page2 | Although there is some understanding of SAP, improving the prognosis of patients with SAP remains a challenge, as the prognosis of patients with SAP is influenced by a variety of factors, and the risk of death in patients with SAP has not yet been adequately identified in clinical practice. |
| Objectives | 3 | State specific objectives, including any prespecified hypotheses | Page3 | Our study aimed to clarify the relationship between baseline PNI levels and the risk of death during hospitalization in SAP patients. |
| Methods | | | |  |
| Study design | 4 | Present key elements of study design early in the paper | Page 3 | This was a retrospective study that consecutively included patients with AIS who attended The Third People's Hospital |
| Setting | 5 | Describe the setting, locations, and relevant dates, including periods of recruitment, exposure, follow-up, and data collection | Page 3 | Demographic and clinical data, including age, sex, hypertension status, type 2 diabetes mellitus (T2DM), COPD, atrial fibrillation (AF) |
| Participants | 6 | (*a*) *Cohort study*—Give the eligibility criteria, and the sources and methods of selection of participants. Describe methods of follow-up  *Case-control study*—Give the eligibility criteria, and the sources and methods of case ascertainment and control selection. Give the rationale for the choice of cases and controls  *Cross-sectional study*—Give the eligibility criteria, and the sources and methods of selection of participants | Page3 | Patients with traumatic brain injury, transient ischemic attack, subarachnoid hemorrhage, acute or chronic infection, autoimmune disease, hematological disorders, or cancer were excluded. |
|  |  | (*b*) *Cohort study*—For matched studies, give matching criteria and number of exposed and unexposed  *Case-control study*—For matched studies, give matching criteria and the number of controls per case |  |  |
| Variables | 7 | Clearly define all outcomes, exposures, predictors, potential confounders, and effect modifiers. Give diagnostic criteria, if applicable | Page 3 | Hypertension was defined as either a blood pressure measurement of ≥140/90 mmHg on separate days or the use of antihypertensive medication. |
| Data sources/ measurement | 8* | For each variable of interest, give sources of data and details of methods of assessment (measurement). Describe comparability of assessment methods if there is more than one group | Page 3-4 | comparisons between two groups were made by t-test or rank-sum test. |
| Bias | 9 | Describe any efforts to address potential sources of bias | Page 4 | Univariate and multivariate logistic regression analyses were used to assess the relationship between the PNI and the risk of experiencing in-hospital mortality in SAP patients. |
| Study size | 10 | Explain how the study size was arrived at |  |  |

Continued on next page

| Quantitative variables | 11 | Explain how quantitative variables were handled in the analyses. If applicable, describe which groupings were chosen and why | Page 3-4 | Measurement information was expressed as mean ± standard deviation or median (interquartile spacing) by normality test, and comparisons between two groups were made by t-test or rank-sum test. |
| --- | --- | --- | --- | --- |
| Statistical methods | 12 | (*a*) Describe all statistical methods, including those used to control for confounding | Page 4 | Univariate and multivariate logistic regression analyses were used to assess the relationship between the PNI and the risk of experiencing in-hospital mortality in SAP patients. |
|  |  | (*b*) Describe any methods used to examine subgroups and interactions |  |  |
|  |  | (*c*) Explain how missing data were addressed |  |  |
|  |  | (*d*) *Cohort study*—If applicable, explain how loss to follow-up was addressed  *Case-control study*—If applicable, explain how matching of cases and controls was addressed  *Cross-sectional study*—If applicable, describe analytical methods taking account of sampling strategy |  |  |
|  |  | (*e*) Describe any sensitivity analyses |  |  |
| Results | | | | |
| Participants | 13* | (a) Report numbers of individuals at each stage of study—eg numbers potentially eligible, examined for eligibility, confirmed eligible, included in the study, completing follow-up, and analysed | Page 4 | The mean age of the 336 SAP patients was 61 ± 13 years, with 250 males (74.4%) and 86 females (25.6%), and in-hospital mortality occurred in 30 SAP patients. |
|  |  | (b) Give reasons for non-participation at each stage |  |  |
|  |  | (c) Consider use of a flow diagram |  |  |
| Descriptive data | 14* | (a) Give characteristics of study participants (eg demographic, clinical, social) and information on exposures and potential confounders | Page 4 | There was a statistically significant difference in Age, COPD, AF, BMI, DBP, LYM, ALB, and RBG among SAP patients with in-hospital and non-in-hospital mortality. |
|  |  | (b) Indicate number of participants with missing data for each variable of interest |  |  |
|  |  | (c) *Cohort study*—Summarise follow-up time (eg, average and total amount) |  |  |
| Outcome data | 15* | *Cohort study*—Report numbers of outcome events or summary measures over time |  |  |
|  |  | *Case-control study—*Report numbers in each exposure category, or summary measures of exposure | Page 4 | The mean age of the 336 SAP patients was 61 ± 13 years, with 250 males (74.4%) and 86 females (25.6%), and in-hospital mortality occurred in 30 SAP patients. |
|  |  | *Cross-sectional study—*Report numbers of outcome events or summary measures |  |  |
| Main results | 16 | (*a*) Give unadjusted estimates and, if applicable, confounder-adjusted estimates and their precision (eg, 95% confidence interval). Make clear which confounders were adjusted for and why they were included | Page 4 | which showed that BMI (OR:0.858, 95%CI: 0.739-0.995, P=0.043), RBG (OR: 1.079, 95% CI: 1.008-1.154, P=0.027), PNI ≥45 (OR: 0.251,95% CI: 0.105- 0.601, P=0.002) were independent influences on in-hospital mortality in SAP patients (Table3). |
|  |  | (*b*) Report category boundaries when continuous variables were categorized |  |  |
|  |  | (*c*) If relevant, consider translating estimates of relative risk into absolute risk for a meaningful time period |  |  |

Continued on next page

| Other analyses | 17 | Report other analyses done—eg analyses of subgroups and interactions, and sensitivity analyses | Page 4 | PNI has a greater predictive value than any single indicator, and it also possesses a higher sensitivity and/or specificity |
| --- | --- | --- | --- | --- |
| Discussion | | | | |
| Key results | 18 | Summarise key results with reference to study objectives | Page 4 | Our study found that baseline PNI levels at admission in SAP patients were significantly lower in the in-hospital mortality group. |
| Limitations | 19 | Discuss limitations of the study, taking into account sources of potential bias or imprecision. Discuss both direction and magnitude of any potential bias | Page 6 | It is important to note that this study has several limitations. Because this study was conducted at a single center, it lacks external validation. |
| Interpretation | 20 | Give a cautious overall interpretation of results considering objectives, limitations, multiplicity of analyses, results from similar studies, and other relevant evidence | Page 6 | We believe that PNI, as a nutritional indicator calculated by a simple laboratory test, can be used for the assessment of short-term prognosis in patients with SAP. |
| Generalisability | 21 | Discuss the generalisability (external validity) of the study results | Page 6 | Timely interventions targeting high-risk patients may reduce the incidence of SAP and ultimately improve patient regression. |
| Other information | |  | | |
| Funding | 22 | Give the source of funding and the role of the funders for the present study and, if applicable, for the original study on which the present article is based | Page 6 | None |

*Give information separately for cases and controls in case-control studies and, if applicable, for exposed and unexposed groups in cohort and cross-sectional studies.

**Note:** An Explanation and Elaboration article discusses each checklist item and gives methodological background and published examples of transparent reporting. The STROBE checklist is best used in conjunction with this article (freely available on the Web sites of PLoS Medicine at http://www.plosmedicine.org/, Annals of Internal Medicine at http://www.annals.org/, and Epidemiology at http://www.epidem.com/). Information on the STROBE Initiative is available at www.strobe-statement.org.
